# Untangling human milk oligosaccharides and infant gut microbiome

**DOI:** 10.1016/j.isci.2021.103542

**Published:** 2021-12-01

**Authors:** Andrea C. Masi, Christopher J. Stewart

**Affiliations:** 1 Translational and Clinical Research Institute, Faculty of Medical Sciences, Newcastle University, 3rd Floor Leech Building, Newcastle NE2 4HH, UK

**Keywords:** Nutrition, Microbiology, Microbiome

## Abstract

The developing gut microbiome in infancy plays a key role in shaping the host immune system and metabolic state, and human milk is the main factor influencing its composition. Human milk does not only serve to feed the baby, but also to help the new-born adapt to its new environment and microbial exposures. Human milk protects the infant by providing multiple bioactive molecules, including human milk oligosaccharides (HMOs), which are the third most abundant solid component after lipids and lactose. The infant is unable to digest HMOs, so they reach the small and large intestines intact where they have many roles, including acting as prebiotics. *Bifidobacterium* spp. are the main, but not the only, commensals equipped with genes for HMO degradation. In this review we will outline the HMOs structures and functions, list the genes needed for their digestion, and describe the main strategies adopted by bacteria for their utilization.

## Graphical abstract

**Figure:**
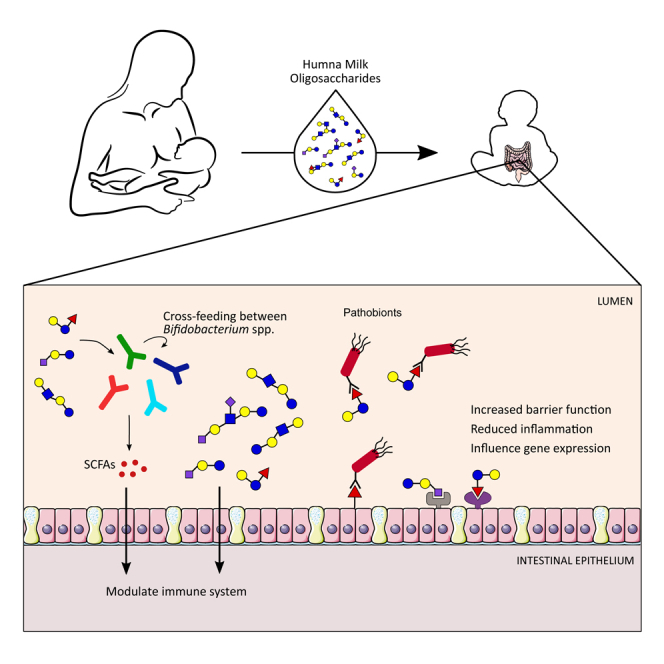

## Introduction

The central role of microorganisms in shaping human health has long been established. Various body sites are colonized by specific microbial communities, which can provide protection from potential pathogenic organisms and participate in host homeostasis (Pflughoeft and Versalovic, 2012). The collection of microorganisms inhabiting the human body is referred to as the microbiome and is composed of bacteria, fungi, archaea, viruses, and bacteriophages. The gastrointestinal tract harbors the densest microbial community in the human body, with up to 10^14^ bacteria (Sender et al., 2016), and represents a vast interface where the immune system interacts with the outside world. Indeed, the intestinal tract hosts a large number of immune cells collectively known as gut-associated lymphoid tissue (GALT), accounting for almost 70% of the entire immune system (Jung et al., 2010).

The gut microbiome participates in human health by digesting food and fibers, producing essential vitamins and amino acids, and by competing with pathobionts, thus helping to prevent potential infections (Jandhyala et al., 2015). A balanced gut-microbiome interaction is essential for host health, and it is shaped from early life (Rautava, 2016; Rodriguez et al., 2015). The gut microbiome composition in the infant has been associated with many later outcomes, including asthma (Fujimura et al., 2016), obesity (Kalliomäki et al., 2008), and celiac disease (Olivares et al., 2018). The human body is considered sterile in the womb, and so microbial colonization starts at birth when the infant is exposed to a diverse array of viable microorganisms. The infant gut is initially colonized by aerobic and facultative anaerobic bacteria, followed by establishment of a more anaerobic community (Palmer et al., 2007; Rodriguez et al., 2015). The determinants of succession are not completely understood and likely represent a combination of location seeding different pioneering and succession species, which reduce oxygen levels over the initial weeks, supporting strict anaerobes (Ferretti et al., 2018).

Many factors cooperate in the microbiome establishment, and mode of delivery (vaginal vs C-section), type of feeding (breast milk vs infant formula), antibiotic administration, environment, and exposure to siblings and pets are among key ones (Rautava, 2016; Rodriguez et al., 2015). However, infant feeding has been found to be the most important contributor to microbiome development (Stewart et al., 2018). Human milk is a complex biofluid, which not only provides nourishment to the new-born, but also helps the immature body cope with foreign environmental stimuli and the microorganisms it first encounters (Ballard and Morrow, 2013). The microbial shaping effect is driven through various mechanisms, including by directly providing potential colonizers from the breast milk microbiome, immune factors (e.g., secretory IgA, antimicrobial peptides, and proteins), and human milk oligosaccharides (HMOs) (Granger et al., 2021). Defining a healthy infant gut microbiome is challenging, but microbial communities rich in *Bifidobacterium* spp. have been associated with positive outcomes and lower risk of various pathologies (Fujimura et al., 2016; Kalliomäki et al., 2008; Stewart et al., 2017), although ecosystem services framework to define dysbiosis have been proposed (Duar et al., 2020b). Colonization of the infant gut by this genus is known to be influenced by type of feeding, largely because of the prebiotic effect exerted by HMOs (Berger et al., 2020; Lawson et al., 2019; Vatanen et al., 2018). Indeed *Bifidobacterium* spp. are the most common HMO utilizers, but not the only ones. In this review, we will outline HMOs structures and functions, what enzymes are needed for their digestion, and what strategies bacteria have developed for their consumption.

## Human milk oligosaccharides composition and structures

HMOs are a family of structurally complex unconjugated glycans characteristic of human milk, which are involved in the modulation of epithelium (He et al., 2016; Lane et al., 2013; Natividad et al., 2020; Wu et al., 2019), immune system (Bode et al., 2004; Eiwegger et al., 2004), and microbiome (Borewicz et al., 2020; Lawson et al., 2019). HMOs represent the third most abundant solid component in human milk after lactose and lipids, with a concentration of 9–24 g/L, which usually exceeds the quantity of total proteins (Bode, 2012; Zuurveld et al., 2020). More than 200 structurally diverse HMOs have been reported, although 20–25 of them are expressed in appreciable quantities and account for >95% of total HMOs. Interest in HMOs in health and disease has gained increased interest, in part, owing to advances in techniques applied for their analysis (Smilowitz et al., 2014; van Leeuwen, 2019). High-pressure liquid chromatography coupled with online fluorescence detection (HPLC-FL) and liquid chromatography mass spectrometry (LCMS) are frequently used in HMO studies, where relative or absolute abundances of HMOs can be determined with these methods (Smilowitz et al., 2014; van Leeuwen, 2019).

**Figure 1:**
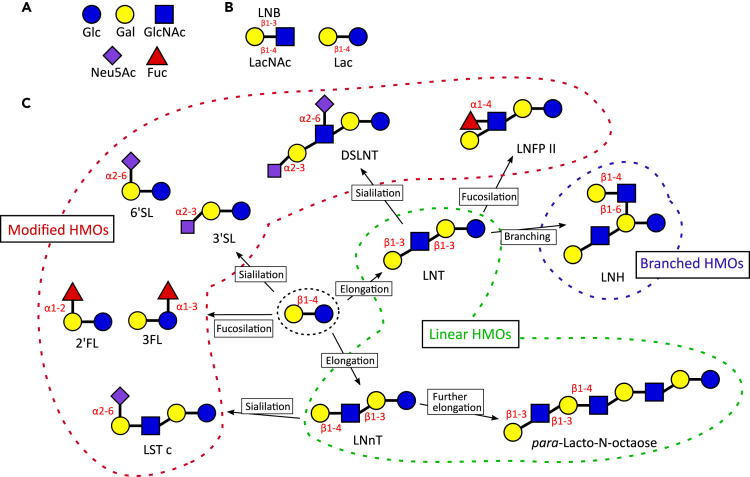
Human milk oligosaccharides structures (A) Monosaccharides. (B) disaccharides composing HMOs. (C) Examples of type of linkages and modification characterizing HMOs. Glc, glucose; Gal, galactose; GlcNAc, N-acetylglucosamine; Neu5Ac, N-acetylneuraminic acid; Fuc, fucose; LNB, lacto-N-biose; LacNAc, N-acetyllactosamine; Lac, lactose; HMOs, human milk oligosaccharides; 2’FL; 2′-fucosyllactose; 3FL, 3-fucosyllactose; LNnT, lacto-N-neotetraose; 3′SL, 3′-sialyllactose; 6′SL, 6′-sialyllactose; LNT, lacto-N-tetraose; LNFP II, lacto-N-fucopentaose II; LST c, sialyl-LNT c; LNH, lacto-N-hexaose; DSLNT, disialyllacto-N-tetraose.

HMOs derive from the arrangement of five monosaccharides: glucose (Glc), galactose (Gal), N-acetylglucosamine (GlcNAc), fucose (Fuc), and sialic acid (N-acetylneuraminic acid (Neu5Ac)) (Figure 1A). GlcNAc and Gal can form two different disaccharides: lacto-N-biose (LNB) in case of β1-3 linkage, N-acetyllactosamine (LacNAc) with β1-4 linkage (Figure 1B). Every HMO is composed by a lactose molecule at the reducing end and a variable number of LNB (type 1 chain) and LacNAc (type 2 chains) (Figure 1C). The lactose molecule is initially elongated with one of the two disaccharides through β1-3 or β1-6 linkage. Although the addition of LNB terminates the chain and is not affected by additional modifications, LacNAc can be further elongated with the introduction of supplementary disaccharides. Branched molecules are formed when the disaccharide is added with a β1-6 linkage. The resulting HMOs can then be modified by the addition of Fuc (α1-2, α1-3, and α1-4 linkage) and/or Neu5Ac (α2-3, α2-6 linkage) (Bode, 2012; Zuurveld et al., 2020).

Although the more abundant HMOs are expressed in comparable amounts between mothers, each woman will produce only a subset of the possible structures, leading to an oligosaccharide composition specific to each mother (Bode, 2012). Although the synthesis of the HMOs relies on many glycosyl-transferases specific for the various monomers, the HMO composition depends on the genetic profile of the mother determining the activity of two fucosyl-transferases. Secretor women express the *Se* gene encoding for an active α1-2-fucosyltransferase FUT2, and Lewis positive women carry a *Le* gene encoding for an active α1-3-fucosyltransferase FUT3. Secretor mothers will be characterized by a breast milk with high concentration of α1-2-fucosylated HMOs (e.g., 2′-fucosyllactose - 2′FL), while the presence of an active *Le* gene is associated with enrichment in α1-4-fucosylated HMOs (e.g., lacto-N-fucopentaose (LNFP) II - LNFP II). *Le*-negative and *Se*-negative women, thus women not expressing active enzymes encoded by these two gene loci, will still have low concentrations of α1-2 and α1-4-fucosylated HMOs, suggesting that other fucosyl-transferases might be involved, even though with low activity (Bode, 2012; Zuurveld et al., 2020).

## Human milk oligosaccharides in infants' health

**Figure 2:**
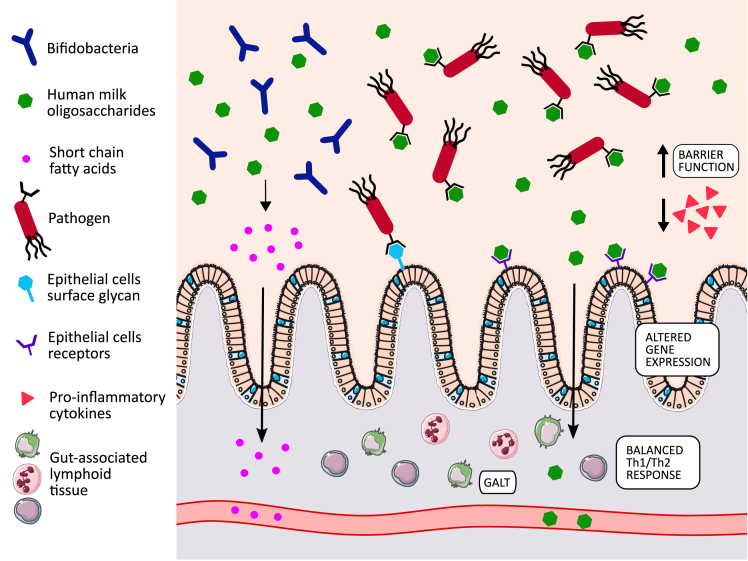
Mechanisms mediated by human milk oligosaccharides and influencing infant health

HMOs are considered prebiotics because being indigestible to the infant, they reach the gut intact (Engfer et al., 2000) where they promote the growth of potentially beneficial bacteria (Figure 2). They are mainly digested by *Bifidobacterium* spp., but can also be used by other bacteria including species within the *Bacteroides* genus (Yu et al., 2013). HMOs protect the infant gut from potential pathogen colonization, not only promoting beneficial bacteria colonization to occupy available niches, but also by directly acting as antiadhesive antimicrobials (Bode, 2012; Zuurveld et al., 2020) (Figure 2). Indeed, they are able to coat pathogens, preventing their adhesion to epithelial surfaces and thus reducing risk of infection, as reported for *E. coli* (Wang et al., 2020) and *Campylobacter jejuni* (Ruiz-Palacios et al., 2003). Such action as decoy receptors is made possible by their resemblance to cell surface glycans which are used by pathogenic species during the infection process (Zuurveld et al., 2020). However, it is notable that specific HMOs are also able to increase infectivity of certain neonatal rotavirus strains, highlighting the need for further work (Ramani et al., 2018).

HMOs can directly interact with and modulate the immune and gastrointestinal systems (Zuurveld et al., 2020) (Figure 2). Sialylated HMOs have been reported to influence lymphocyte maturation and modulate a low-level immunity by shaping the immune system toward a regulatory type Th1 response (Eiwegger et al., 2004). Acidic HMOs were also able to modulate and lower monocyte, lymphocyte, and neutrophil adhesion to endothelial cells, where excessive leukocyte infiltration can cause major tissue injury in inflammatory diseases (Bode et al., 2004). Beyond the gut it has been further reported that ∼1% of HMOs can be absorbed through the gut into the systemic circulation, giving rise to the possible impacts on modulating systemic immunity (Bode, 2012). At the level of intestinal tissue, HMOs are able to modulate the development and maturation of the gut. As reported by Lane et al. (2013), exposure of HT29 cells to HMOs impacted the gene expression profile, with modulation of cytokines, chemokines, and cell surface receptors (Lane et al., 2013). In another study, epithelial cells exposed to HMOs produced lower levels of pro-inflammatory mediators, whereas cytokines involved in tissue repair and homeostasis were increased (He et al., 2016). Intestinal barrier function can also be modulated by HMOs, with reduced gut permeability observed after treatment with these glycans (Natividad et al., 2020; Wu et al., 2019).

## Human milk oligosaccharides enzymatic degradation

**Figure 3:**
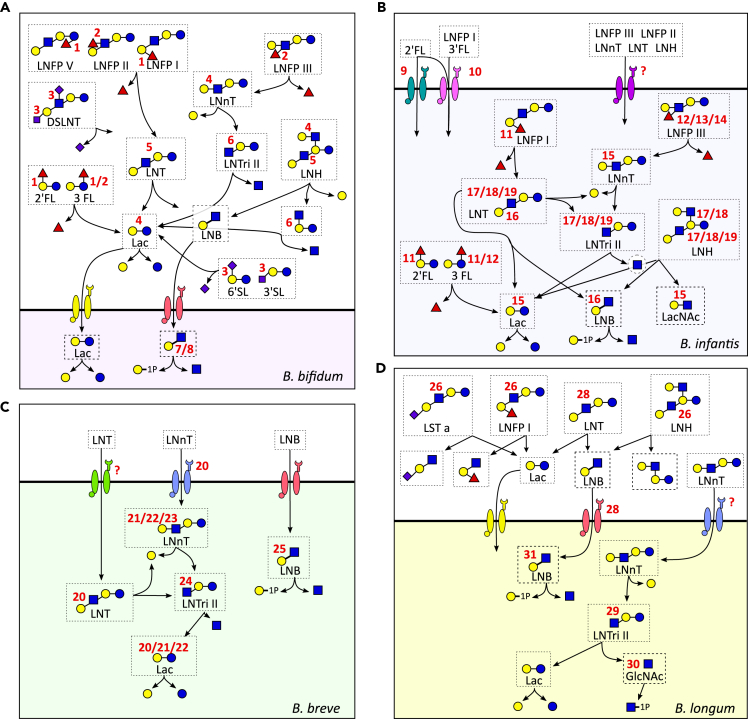
Schematic representation of the main human milk oligosaccharides metabolized by *B. bifidum* (A), *B. longum* subsp. *infantis* (B), *B. breve* (C), and *B. longum* subsp. *longum* (D). Proteins responsible for cleavage of the various bonds or transport of the oligosaccharides are represented by the red numbers; numbers refer to the gene list reported in Table 1. Where no protein harboring the specific activity has been identified, a “?” has been added. In other instances where no red symbol is reported, the reaction is mediated by homologues of known genes. 2′FL, 2′-fucosyllactose; 3FL, 3-fucosyllactose; LNnT, lacto-N-neotetraose; 3′SL, 3′-sialyllactose; 6′SL, 6′-sialyllactose; LNT, lacto-N-tetraose; LNFP I, lacto-N-fucopentaose I; LNFP II, lacto-N-fucopentaose II; LNFP III, lacto-N-fucopentaose III; LNFP V, lacto-N-fucopentaose V; LST a, sialyllacto-N-tetraose a; LNH, lacto-N-hexaose; DSLNT, disialyllacto-N-tetraose; Lac, lactose; LNB, lacto-N-biose; LNTri II, lacto-N-triose II; LacNAc, N-acetyllactosamine; GlcNAc, N-acetylglucosamine.

For HMO utilization, the single monosaccharides need to be released, and the breakdown of each linkage requires specific enzymes that exist in microbes (Table 1). To be able to utilize the core structure, the modifications are often removed first (Ashida et al., 2009) (Figure 3). The enzymes responsible for fucose release are named fucosidases, and two types are present: 1,2-α-L-fucosidase acting on α-1,2 linkage and 1,3-1,4-α-L-fucosidase acting on fucose added with α-1,3 and α-1,4 linkages. The first enzyme will act mainly on 2′FL, LNFP I (Katayama et al., 2004), and the second on 3FL, LNFP II, and LNFP III (Ashida et al., 2009). Sialidases on the other hand are enzymes responsible for the liberation of Neu5Ac from the core structure by acting on the α-2,3 and α-2,6 linkages (Kiyohara et al., 2011). Bacteria can further metabolize Fuc and Neu5AC released from the core structures (Brigham et al., 2009; Bunesova et al., 2016; Salli et al., 2021).

**Table 1:** Genes characterized in papers cited in this review

	*Bifidobacterium* strain	Protein/enzyme type	Abbreviation or gene locus	Enzymatic activity	Preferred HMO substrates	Reference
1	*B. bifidum* JCM1254	1,2-α-L-fucosidase	AfcA	Extracellular	2′FL, LNFP I, limited activity on 3′FL and LNFP V	(Katayama et al., 2004)
2	*B. bifidum* JCM1254	1,3-1,4-α-L-fucosidase	AfcB	Extracellular	3′FL, LNFP II, LNFP III	(Ashida et al., 2009)
3	*B. bifidum* JCM1254	Exo-α-sialidase	SiaBb2	Extracellular	3′SL, DSLNT, 6′SL	(Kiyohara et al., 2011)
4	*B. bifidum* JCM1254	β-galactosidase	BbgIII	Extracellular	LacNAc, LNnT, LNH, Lac	(Miwa et al., 2010)
5	*B. bifidum* JCM1254	Lacto-N-biosidase	LnbB	Extracellular	LNT, LNH	(Wada et al., 2008)
6	*B. bifidum* JCM1254	β-N-Acetylglucosaminidase	BbhI	Extracellular	LNTri	(Miwa et al., 2010)
7	*B. bifidum* JCM1254	GNB/LNB phosphorylase	LnpA1	Intracellular	LNB/GNB	(Nishimoto and Kitaoka, 2007b)
8	*B. bifidum* JCM1254	GNB/LNB phosphorylase	LnpA2	Intracellular	LNB/GNB	(Nishimoto and Kitaoka, 2007b)
9	*B. longum* subsp. *infantis* ATCC 15697	Transporter SBP	FL1-BP	–	2′FL	(Sakanaka et al., 2019b)
10	*B. longum* subsp. *infantis* ATCC 15697	Transporter SBP	FL2-BP	–	2′FL, 3′FL, LDFT, LNFP I	(Sakanaka et al., 2019b)
‘11	*B. longum* subsp. *infantis* ATCC 15697	α-L-fucosidase	AfcA	Intracellular	LNFP I, 2'FL, 3'FL	(Sela et al., 2012)
12	*B. longum* subsp. *infantis* ATCC 15697	1,3-1,4-α-L-fucosidase	AfcB	Intracellular	LNFP III, 3'FL	(Sela et al., 2012)
13	*B. longum* subsp. *infantis* ATCC 15697	1,3-1,4-α-L-fucosidase	Blon_0248	Intracellular	LNFP III	(Sela et al., 2012)
14	*B. longum* subsp. *infantis* ATCC 15697	α-L-fucosidase	Blon_0426	Intracellular	LNFP III	(Sela et al., 2012)
15	*B. longum* subsp. *infantis* ATCC 15697	β-galactosidase	Bga2A	Intracellular	Lac, LacNAc, LNnT	(Yoshida et al., 2011)
16	*B. longum* subsp. *infantis* ATCC 15697	LNT β-1,3-Galactosidase	Bga42A	Intracellular	LNT, LNB	(Yoshida et al., 2011)
17	*B. longum* subsp. *infantis* ATCC 15697	β-N-Acetylglucosaminidase	Blon_0459	Intracellular	LNT, LNH, LNTri	(Garrido et al., 2012)
18	*B. longum* subsp. *infantis* ATCC 15697	β-N-Acetylglucosaminidase	Blon_0732	Intracellular	LNT, LNH, LNTri	(Garrido et al., 2012)
19	*B. longum* subsp. *infantis* ATCC 15697	β-N-Acetylglucosaminidase	Blon_2355	Intracellular	LNT, LNH, LNTri	(Garrido et al., 2012)
20	*B. breve* UCC2003	Transporter SBP	NahS	–	LNnT	(James et al., 2016)
21	*B. breve* UCC2003	β-galactosidase	LntA	Intracellular	LNT, LNnT, Lac	(James et al., 2016)
22	*B. breve* UCC2003	β-galactosidase	LacZ2	Intracellular	LNnT, Lac	(James et al., 2016)
23	*B. breve* UCC2003	β-galactosidase	LacZ6	Intracellular	LNnT, Lac	(James et al., 2016)
24	*B. breve* UCC2003	β-N-Acetylglucosaminidase	NahA	Intracellular	Lacto-N-triose	(James et al., 2016)
25	*B. breve* UCC2003	GNB/LNB phosphorylase	LnbP	Intracellular	LNB	(James et al., 2016)
26	*B. longum* subsp. *longum* JCM1217	Lacto-N-biosidase	LnbX	Extracellular	LNT, LNH, LNFP I, LST a	(Sakurama et al., 2013)
27	*B. longum* subsp. *longum* JCM1217	Chaperone for LnbX	LnbY	Extracellular	–	(Sakurama et al., 2013)
28	*B. longum* subsp. *longum* JCM1217	Transporter SBP	GL-BP	–	LNB, GNB	(Suzuki et al., 2008)
29	*B. longum* subsp. *longum* JCM1217	β-N-Acetylglucosaminidase	BLLJ_1391	Intracellular	LNTri	(Honda et al., 2013)
30	*B. longum* subsp. *longum* JCM1217	N-acetylhexosamine 1-kinase	NahK	Intracellular	GlcNAc/GalNAc	(Kitaoka et al., 2005)
31	*B. longum* subsp. *longum* JCM1217	GNB/LNB phosphorylase	LnpA	Intracellular	LNB/GNB	(Kitaoka et al., 2005)

Protein abbreviation was reported where available, otherwise gene locus was used.

After modifications are removed, the core structure can be digested. Two enzymes act on Gal: β-1,3-galactosidase which acts mainly on LNT, but also on β-1,4 linkage of Lac, LNB and lacto-N-neotetraose (LNnT) (James et al., 2016; Yoshida et al., 2011); β-1,4-galactosidases act on the β-1,4 linkage found in type 2 chains and Lac (James et al., 2016; Miwa et al., 2010). Further disassembling the HMOs structures, the bond between GlcNAc and Gal is cleaved by β-N-acetylglucosaminidases. Various enzymes of this type have been reported in literature, each having their own preferences for the specific HMOs targeted and some of which being able to act on both β-1,3 and β-1,6 linkages equally, whereas others having a preference on specific linkage type (Honda et al., 2013; James et al., 2016; Miwa et al., 2010). In certain cases, the first and outer Gal residue needs to be removed, before the enzyme can free the GlcNAc (Garrido et al., 2012). Finally, the enzyme Lacto-N-biosidase can act on lacto-N-tetraose (LNT) generating LNB and Lac, which can be further metabolized (Sakurama et al., 2013; Wada et al., 2008).

## Methodologies for identification of HMO utilization genes

**Figure 4:**
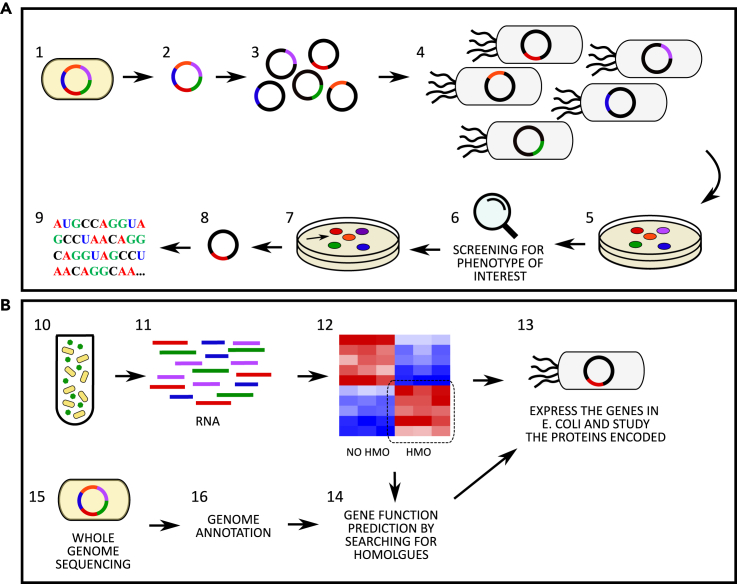
Methodologies for the identification of human milk oligosaccharides utilisation genes (A) Approach involving genomic library formation. The bacterium of interest (1) is lysed, and the genome is retrieved (2). The genome is randomly fragmented, and the fragments are inserted in a cloning vector (3), which are then transfected in the host bacterium (4), creating the genome library (5). The clones composing the genome library are then screened for the identification of the phenotype of interest (6) and the colony harboring such a function (7) is retrieved. The vector is re-isolated from the identified clone (8) and the gene sequence is determined. (B) Approaches relying on sequencing methods. The bacterium of interest is grown in media containing HMOs as the sole carbon source (10), the RNA is extracted (11) and the transcriptome profile determined. The function of the genes upregulated in media containing HMOs vs the sugar of interest is subsequently determined by inserting it in a vector and expressing it in a host bacterium (13), or by predicting its function searching for homologues (14). Alternatively, the sequencing of the bacterial genome is performed (15), the genome is annotated (16) and the gene function is predicted by searching for homologues (14). The gene function can be further studied by expressing the gene in a host bacterium, as described above (13).

Generation of genomic libraries has often been applied for the discovery and characterization of genes implied in HMOs degradation (Katayama et al., 2004; Møller et al., 2001; Sakurama et al., 2013). This approach consists of various steps: initially the whole genome of the bacterium of interest is randomly fragmented; the DNA fragments obtained are inserted in an appropriate vector, and the collection of vectors obtained is transfected in a suitable bacterial host (usually *E. coli*) (Clark and Pazdernik, 2013) (Figure 4A). The collection of clones obtained is the genomic library, which can subsequently be screened for the target function by observation of the phenotype of interest (Clark and Pazdernik, 2013). For instance, to determine novel genes involved in fucose utilization, Katayama et al. (2004) lysed each transformed colony, and the cell content was incubated with 2′FL. The reaction mixture was then analyzed using a thin-layer chromatography to identify the colonies harboring the function desired (Katayama et al., 2004). After selecting the clones showing the acquired phenotype, retrieval of the gene sequence can be performed by sequencing the fragment inserted in the vector (Katayama et al., 2004).

Transcriptome profiling, utilizing RNA-sequencing (Garrido et al., 2016) or microarrays (James et al., 2016), has also been used (Figure 4B). The bacterial species are initially grown in the specific substrate of interest, the RNA is subsequently extracted, converted to cDNA, and the transcription profile defined. To identify induced genes associated with the substrate metabolism, a known and well characterized condition is used as control (Garrido et al., 2016; James et al., 2016). For instance, Garrido et al. (2016) grew *B. longum* in lactose and performed transcriptome analysis to identify HMO utilization genes in this species (Garrido et al., 2016). Genes with significantly elevated expression by exposure to HMOs or related structures can then be further characterized in subsequent experiments, such as purification and characterization of the protein encoded (Garrido et al., 2016; James et al., 2016). The putative role exerted by the identified genes can also be determined *in silico* by comparing the gene sequence and/or protein sequence with known genes and proteins stored in specific databases (James et al., 2016). This process is also central to the whole genome sequencing approach (Ashida et al., 2009; LoCascio et al., 2010; Miwa et al., 2010; Sela et al., 2008) (Figure 4B). In the first step, the bacterial genome sequence is annotated, which consists in identification of genes, promoters, rRNA genes, untranslated regions, and pseudogenes (Beckloff et al., 2012). The function of the genes is then identified by comparing their sequence identity towards genes of known function stored in publicly available databases. Owing to declining sequencing costs, the number of genomes deposited in the dedicated databases is increasing, allowing the study of the conservation of HMO utilization genes and gene clusters in bacteria families, genera, species, and strains (Kitaoka et al., 2005).

The genomic library screening and transcriptome profiling approaches allow the identification of novel genes, as they do not rely on characterized genes reported in the databases. On the contrary, the whole genome sequencing method can only find homologous of known HMO utilization genes in uncharacterized bacterial species and strains. Thus, complementing the different techniques is central to expanding the collection of the well characterized genes and investigating their distribution and conservation in bacteria.

### *Bifidobacterium*

The first observation of the enrichment in bifidobacteria in the stool of breast milk fed infants compared to formula fed infants dates back to 1900, through the work of Tissier at the Pasteur Institute (Tissier, 1900). Recent advances in DNA sequencing technologies have further shown the correlation between breast milk ingestion and bifidobacteria colonization, with multiple studies characterizing early life gut microbiome establishment (Berger et al., 2020; Borewicz et al., 2020; Lawson et al., 2019; Vatanen et al., 2018). Bifidobacteria are anaerobic Gram-positive bacteria belonging to the Actinobacteria phylum and are generally considered to be beneficial to the human body through various mechanisms including production of short-chain fatty acids (SCFAs) (Alessandri et al., 2019). The central role of HMOs in explaining breast milk's bifidogenic-effect was discovered in the mid-1900s (György et al., 1954); however, the first studies elucidating the mechanisms and genes involved in their utilization by species belonging to this genus are relatively recent, dating to early 2000 (Katayama et al., 2004; Kitaoka et al., 2005; Møller et al., 2001; Nishimoto and Kitaoka, 2007a; Sela et al., 2008). The ability of *Bifidobacterium* spp. to colonize the human gut differ by the host stage of life, with *B. breve, B. bifidum* and *B. longum* subsp. *infantis* being found mainly in breastfed infants, whereas older subjects usually carry *B. adolescentis* and *B. catenulatum*. Only *B. longum* subsp. *longum* has been reported to colonize the human gut throughout life (Alessandri et al., 2019). Such differences can be explained, in part, by the bacteria’s capability to utilize HMOs, which can greatly vary between different species, subspecies, and strains.

Two different strategies for HMOs digestion have been reported: *B. longum* subsp. *infantis, B. breve,* and *B. longum* tend to internalize the oligosaccharides through ATP-binding cassette (ABC) transporters and digest the sugar structure internally (Asakuma et al., 2011; Garrido et al., 2016; Møller et al., 2001; Sakanaka et al., 2019b; Sela et al., 2008) (Figures 3B–3D). On the contrary, *B. bifidum* is equipped with extracellular glycosidases (Ashida et al., 2009; Katayama et al., 2004; Møller et al., 2001; Wada et al., 2008), which therefore act on the HMO linkages outside of the cell and release the monosaccharides and disaccharides (LNB and Lac) in the surrounding environment, which can either be left for the growth of other bacteria or transported internally to be metabolized inside the cell (Asakuma et al., 2011; Gotoh et al., 2018; Kiyohara et al., 2011; Miwa et al., 2010; Nishimoto and Kitaoka, 2007b; Suzuki et al., 2008) (Figure 3A). Extracellular glycosidases have also been found in *B. longum,* but with a lower frequency (Sakanaka et al., 2019a; Sakurama et al., 2013). Likely because of the strategy adopted, *B. bifidum* has been demonstrated to cross-feed other *Bifidobacterium* spp. not equipped for HMO degradation, but able to utilize the released degradants, as reported both *in vitro* (Gotoh et al., 2018) and *in vivo* from infant gut microbiome studies (Tannock et al., 2013).

*B. bifidum* and *B. infantis* strains are the most frequent utilizers of HMOs, and many different structures, modified and non-modified (i.e., structures carrying or not Fuc and/or Neu5Ac), can be digested by these two (sub)species (Garrido et al., 2016; Katayama et al., 2004; LoCascio et al., 2007, 2010; Sela et al., 2008, 2012). On the contrary, *B. longum* and *B. breve* can utilize only LNT, LNB, and LNnT, whereas utilization of modified HMOs has been reported only in a few strains (Asakuma et al., 2011; Thongaram et al., 2017). *B. infantis* is reported to be the most efficient utilizer of HMOs and is able to consume up to 64% of total pooled HMOs, compared to a utilization between 23 and 43% displayed by other species (LoCascio et al., 2007, 2010). Sela et al. (2008) were the first to report a vast HMO-utilization cluster in *B. infantis* comprising 30 genes, some of which are likely subjected to a communal transcriptional regulation (Sela et al., 2008). This cluster comprises 4 glycosidases (a fucosidase, a sialidase, a β-N-acetylglucosaminidase, and a β-galactosidase), 2 ABC transport permeases and associated ATPase, 7 solute binding proteins (SBPs) predicted to bind oligosaccharides (Sela et al., 2008). Other genes implicated in HMO utilization are also found in other positions in the genome. Moreover, a total of 21 copies of family 1 SBPs were found in this subspecies, compared to 10 and 11 found in *B. longum* and *B. adolescentis*, and 6 of the SBPs in the cluster show evolutionary divergence compared to other family 1 SBPs (Sela et al., 2008). These characteristics of *B. infantis* guarantee its potential to utilize many different HMOs applying a strategy of internal hydrolysis and compete in the infant gut. It has to be noted that not all *B. infantis* strains are equipped with the full set of genes reported in strain JCM 1260 (LoCascio et al., 2010), and indeed showed lower capacity of growth in HMOs compared to other *B. infantis* strains (Locascio et al., 2009). However, not all genes involved in HMO degradation have been identified to date. Lawson et al. (2019) isolated bifidobacterial strains that were able to grow on HMOs, but lacking known genes and clusters for their digestion, suggesting the presence of uncharacterized HMO utilization genes (Lawson et al., 2019). This underlines the necessity of further studies, such as the recent work expanding the understanding of genes responsible for HMO utilization in commercial *B. infantis* strains (Duar et al., 2020a).

### *Bacteroides*

Bacterial species belonging to the *Bacteroides* genus are frequent colonizers of the term new-born and adult intestine (Shao et al., 2019; Stewart et al., 2018). Vaginal delivery seems to be the key to colonization of the infant gut by this genus and studies suggest the vertical transfer of *Bacteroides* spp. from the mother’s gut microbiome during the delivery (Shao et al., 2019). *Bacteroides* contain specialized genes clustered in genomic regions referred to as polysaccharide utilization loci (PUL) that provide capacity to break down various complex polysaccharides derived from the diet (Flint et al., 2012) and composing the fungal cell wall as well as host-derived glycans (Temple et al., 2017). Indeed, they can hydrolyze the intestinal mucus, which, being composed of Neu5Ac, Fuc, GlcNAc, Gal, is structurally similar to HMOs, placing *Bacteroides* in a favourable position for utilising milk glycans (Marcobal et al., 2011).

Multiple studies have investigated the ability of *Bacteroides* spp. to utilize HMOs, albeit to a lesser extent compared to bifidobacteria. Strains of *B. fragilis, B. vulgatus, B. thetaiotaomicron,* and *B. caccae* were able to grow on pooled HMOs (Marcobal et al., 2010, 2011). *B. fragilis* grew better and consumed a higher proportion of HMOs when compared to *B. vulgatus*. The two *Bacteroides* spp. also differed in their preference for which HMOs were utilized, with *B. fragilis* preferring non-modified HMOs compared to fucosylated glycans, whereas no difference in HMOs preference was observed for *B. vulgatus*. Involving the same strains, Yu et al. (2013) reported *B. vulgatus* and *B. fragilis* could utilize 2′FL, 3-fucosyllactose (3FL), lactodifucotetraose (LDFT), and 6′-sialyllactose (6′SL); *B. vulgatus* could also metabolize 3′SL (Yu et al., 2013). Growth of a different strain of *B. thetaiotaomicron* was also investigated, and all HMOs mentioned with the exception of LDFT were degraded by this bacterium (Yu et al., 2013). Other different strains of *B. vulgatus, B. fragilis,* and *B. thetaiotaomicron* could grow on 2′FL, 3FL, difucosyllactose (DFLac), and Fuc (Salli et al., 2021). This latter *B. thetaiotaomicron* was demonstrated to partially utilize also LNT, LNnT, LNFP I, LNFP II, LNFP III, and LNFP V (Chia et al., 2020).

The genes involved in HMO utilization in *Bacteroides* spp. were elucidated by Marcobal et al. (2011) (Marcobal et al., 2011). Both *B. fragilis* and *B. thetaiotaomicron* utilized PULs loci involved in mucin degradation, even though the genes activated in the two species were different, suggesting distinct strategies applied in different spp. Although *B. thetaiotaomicron* could efficiently grow on HMOs, it was readily outcompeted by *B. infantis* in an *in vivo* mouse model fed with LNnT (Marcobal et al., 2011). These data suggest that, even though *Bacteroides* spp. are able to opportunistically hydrolyze HMOs thanks to their resemblance to mucin structure, bifidobacteria have evolved genes for the selective utilization of glycan structures exclusive of human milk. This would explain why bifidobacteria can colonize the infant gut at higher relative abundances compared to other HMO utilizing bacteria.

## *Lactobacillus* and other gut commensals

*Bifidobacterium* and *Bacteroides* are not the only colonizers of the infant gut microbiome, and other genera frequently found in this niche include *Lactobacillus, Escherichia, Klebsiella, Enterococcus, Staphylococcus,* and *Clostridium* (Shao et al., 2019; Stewart et al., 2018). Utilization of HMOs from these gut commensals has been reported, even though it is sporadic and often strain-specific (Hoeflinger et al., 2015; Marcobal et al., 2010; Salli et al., 2021; Schwab and Gänzle, 2011; Thongaram et al., 2017; Yu et al., 2013). In this regard, Lactobacilli are the most studied as they are often found in the infant developing intestinal microbiome even if at lower persistence and abundance compared to bifidobacteria (Shao et al., 2019; Stewart et al., 2018). Lactobacilli are also often included in infant probiotic formulations together with bifidobacteria, and have thus received more attention compared to other gut colonizers.

Many infant gut commensals, considered non-HMO utilizers, have been tested for growth on these glycans showing weak or absent utilization. Frequent HMOs used for growth curve experiments include 2′FL, 3FL, and LNnT, which could sustain moderate or little growth for strains of *Clostridium perfringens, Escherichia coli, Enterococcus faecalis, Lactobacillus acidophilus, Lactobacillus casei, Lactobacillus fermentum, Lactobacillus plantarum, Lactobacillus rhamnosus, Lactobacillus salivarius, Staphylococcus epidermidis,* and *Streptococcus thermophilus* (Hoeflinger et al., 2015; Marcobal et al., 2010; Salli et al., 2021; Schwab and Gänzle, 2011; Thongaram et al., 2017; Yu et al., 2013). Strains which cannot utilize HMOs, can often metabolize the building blocks composing their structure, including Fuc, GlcNAc, Neu5Ac, and other monosaccharides and disaccharides which can be released when these sugars are digested (Hoeflinger et al., 2015; Salli et al., 2021; Schwab and Gänzle, 2011; Thongaram et al., 2017). Thus, they might profit from *Bifidobacterium* and *Bacteroides* spp. breaking down the HMOs available and releasing the fragments in the environment. Many homologous of HMO utilization genes have been identified when mining bacterial genomes deposited in the databases (Katayama et al., 2004; Kiyohara et al., 2011; Miwa et al., 2010; Sakurama et al., 2013); however, the enzymatic activity of such genes on HMOs is largely unexplored. Notably, the presence of genes for sugar degradation alone does not guarantee the ability to grow in the presence of HMOs. For instance, *L. casei* BL23 is equipped with an α-fucosidase which can act on 2′FL, but it is unable to grow on this HMO (Rodríguez-Díaz et al., 2011). This inability to grow is likely because of the internal localization of the enzyme and lack of a trans-membrane transporter for 2′FL, further highlighting the necessity to test *in vitro* the actual capability of the bacteria to grow on HMOs.

## HMOs metabolism by-products

One of the mechanisms involved in the health-shaping effect exerted by the gut microbiota is through the interaction between microbial metabolites and the host. Some of these potentially beneficial metabolites are produced through the fermentation of diet indigestible carbohydrates in the intestine, including HMOs in the infant population (Stewart, 2021). SCFAs are among the most studied microbial metabolites, with particular attention given to acetate, butyrate, and propionate. SCFAs represent an energy source for colonocytes and can influence host physiology and immune system (Plaza-Díaz et al., 2018). The mechanisms mediating SCFAs beneficial effects include lowering the pH of the intestinal environment, thus preventing the growth of pathobionts (Alessandri et al., 2019; Zuurveld et al., 2020). SCFAs can also directly influence the intestinal health through increase in mucin production and enhancement of the barrier function at the level of intestinal cells, coupled with modulation of the immune system by promoting a GALT population enriched with regulatory T cells (Alessandri et al., 2019; Zuurveld et al., 2020). Partial absorption and release of SCFAs in the systemic circulation leads to a widespread effect of SCFAs in the human body, including modulation of glucose homeostasis, lipid metabolism, and appetite regulation (Morrison and Preston, 2016). Moreover, decreased colonization by SCFA producing bacteria in early life has been associated with Type 1 diabetes (Vatanen et al., 2018) and allergy (Abrahamsson et al., 2012) onset later in life.

Intestinal SCFA composition evolves during the first months of life, likely mirroring the changes occurring in the gut microbial community. Although acetate is produced by most intestinal bacteria and is indeed the most abundant SCFA in the gut, butyrate and propionate can be produced only by a subpopulation of the gut colonizers (Koh et al., 2016; Morrison and Preston, 2016). Consequence of these differences, acetate can be found in detectable quantities since early life, whereas propionate and butyrate concentrations increase over the first year of life, in parallel with the colonization of bacteria involved in their production (Appert et al., 2020; Nilsen et al., 2020; Tsukuda et al., 2021). Other less studied SCFAs associated molecules, lactate and succinate, and the SCFA formate, were instead higher in the first months of life and decreased until 1 year of age (Tsukuda et al., 2021). Metabolism of fucosylated HMOs by bifidobacteria has been reported to be central to formate production. Bifidobacteria are also able to produce acetate, while they lack the pathway for butyrate and propionate production (Louis and Flint, 2017; Rivière et al., 2016). However, bifidobacterial metabolism of Fuc is able to produce butyrate and propionate precursors which might be utilized by other gut commensals to produce these SCFAs (Bunesova et al., 2016). Work from Frese et al. (2017) supports the correlation between HMOs, *B. infantis* supplementation and SCFAs production (Frese et al., 2017). Breastfed infants supplemented with a commercial *B. infantis* strain showed increased quantities of lactate, acetate, butyrate, and formate, but not propionate, in the stool compared to non-supplemented breastfed infants.

## HMOs synthesis and supplementation

The many beneficial effects exerted by human milk through HMOs and other bioactive molecules are continuously being elucidated. However, for various reasons not all infants receive their mom's milk and providing the best nutritive formulations to these infants are of great importance. Owing to the current difficulties in synthetizing HMOs at industrial levels (Zeuner et al., 2019), infant formulas are often supplemented with prebiotic molecules mimicking the human milk glycans, such as galacto-oligosaccharides and fructo-oligosaccharides (GOS and FOS, respectively) (Zuurveld et al., 2020). Like HMOs, GOS, and FOS are indigestible to the infant, reach the infant’s intestine intact, promote the growth of *Bifidobacterium,* can influence the immune system, and bind pathobionts (Zuurveld et al., 2020). However, they are not naturally present in human milk and thus past and current research aims to widen the range of HMOs available to supplement infant formulas to better mirror the human milk formulation.

Great advances have been, and continue to be, made in our understanding of HMOs synthesis, but only a few HMOs can currently be produced in quantities sufficient for their supplementation in infant formulas (Zeuner et al., 2019). The first randomized multicentre trial testing HMOs supplementation was performed in the USA using 2′FL. This trial did not show an impact on infant growth rate compared to non-supplemented formulas and breast milk fed infants (Marriage et al., 2015). Notably, 2′FL was well tolerated, and no adverse events were reported. In a subset of the cohort, plasma cytokines concentrations were also measured and infants receiving formula supplemented with 2′FL displayed lower pro-inflammatory cytokine and TNF-alfa concentrations in the plasma when compared to non-supplemented formula, whereas no difference was observed with respect to breastfed infants (Goehring et al., 2016). In a different randomized trial conducted in Belgium and Italy, supplementation with both 2′FL and LNnT again showed HMO supplementation was well-tolerated and did not alter the infant growth (Puccio et al., 2017). Administration of these HMOs also shifted the developing gut microbiome toward a composition more similar to breastfed infants, and decreased medication use was reported compared to non-supplemented formula fed infants (Berger et al., 2020). Very recently, a European trial involving multiple centers across different countries, reported the outcome of supplementation of 5 HMOs - 2′FL, 3FL, LNT, 3′-sialyllactose (3′SL), and 6′SL (Parschat et al., 2021). Overall, the 5-HMOs blend was well-tolerated by term infants enrolled and no difference in growth rates were observed when compared to non-supplemented formula (Parschat et al., 2021).

## Future perspectives

Even though the HMOs tested in the mentioned trials did not show any negative effects, additional studies including larger numbers of infants involved from multiple countries around the world are needed. Indeed, such studies looked at short-term outcomes, and no information on long-term impact is yet available. HMO composition is known to vary during lactation, by geographical location, secretor status of the mother, term and preterm delivery, ethnicity, and other variables (Austin et al., 2019; Azad et al., 2018; Gabrielli et al., 2011; McGuire et al., 2017). These factors will need to be considered to tailor the supplementation guidelines for different populations, and at different infant ages. As the technology improves, more HMOs will potentially be available for large scale distribution, and the evaluation of the effect of each distinct glycan will be needed. For instance, LNT increased infectivity of a neonatal rotavirus strain (Ramani et al., 2018), whereas disialyllacto-N-tetraose (DSLNT) has been associated to protection against necrotizing enterocolitis in preterm infants (Autran et al., 2018; Masi et al., 2020). However, the mechanism behind the putative protective effect of DSLNT is still unknown and might be driven by its direct modulatory effects exerted on the host immune system and/or intestinal epithelium, and thus be microbiome-independent. Targeted screening of HMO composition of donor human milk might be of interest for the preterm infant population, to ensure that milk high in DSLNT is administered to preterm infants at higher risk of necrotizing enterocolitis. HMO utilization as supplements and therapeutics will likely be an important use in the future, but more data on their role and the underlying mechanisms is needed.

## Abbreviations

2′FL: 2′-fucosyllactose
3FL: 3-fucosyllactose
ABC transporter: ATP-binding cassette transporter
DSLNT: disialyllacto-N-tetraose
FOS: fructo-oligosaccharides
Fuc: fucose
Gal: galactose
GALT: gut-associated lymphoid-tissue
Glc: glucose
GlcNAc: N-acetylglucosamine
GOS: galacto-oligosaccharides
HMO: human milk oligosaccharide
LacNAc: N-acetyllactosamine
LDFT: lactodifucotetraose
LNB: lacto-N-biose
LNFP: lacto-N-fucopentaose
LNH: lacto-N-hexaose
LNnT: lacto-N-neotetraose
LNT: lacto-N-tetraose
LNTri: lacto-N-triose
LST: sialyl-LNT
Neu5Ac: N-acetylneuraminic acid
PUL: polysaccharide utilization loci
SCFA: short-chain fatty acids
